# Supplementary material for: Elastocapillary deformation of thin elastic ribbons in 2D foam columns
Source: Soft Matter. 2022 Jan 31;18(12):2325–31. doi: 10.1039/d1sm01687c (PMC9466004; doi:10.1039/d1sm01687c)
Supplement: SM-018-D1SM01687C-s001 [file SM-018-D1SM01687C-s001.pdf]

Electronic Supplementary Material (ESI) for Soft Matter.
This journal is © The Royal Society of Chemistry 2022

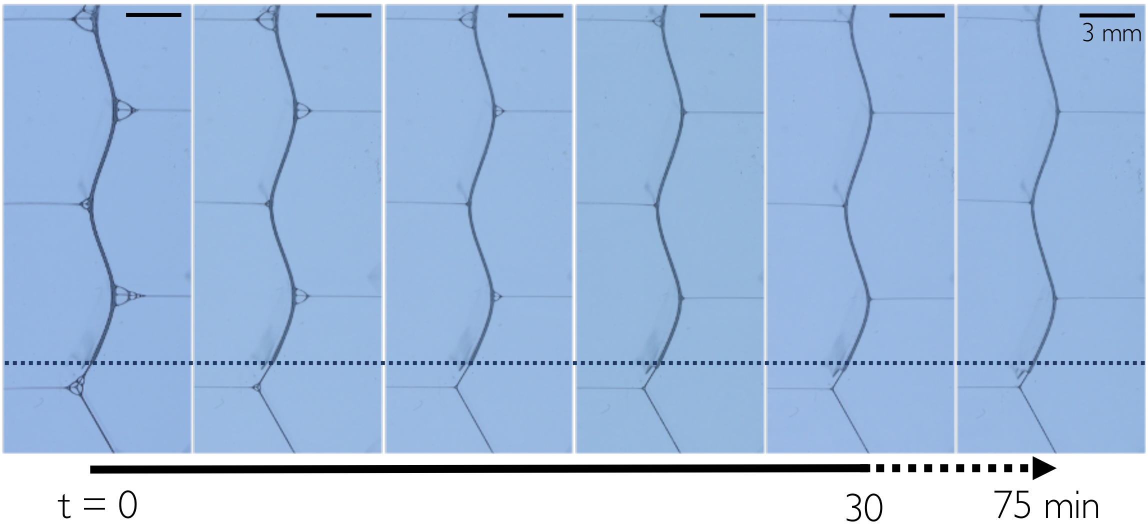

Figure S1: Time evolution over 75 minutes of a quasi-2D foam/ribbon system. It is important to let the system drain for 30 minutes to allow time for soap liquid to drain, the small stray bubbles to disappear and the ribbon to slide down. The dotted line marks the end of the ribbon at t=0, we notice that at t=30 min the bottom of the ribbon slides below this line showing the sliding of the ribbon. After this time, the system is stable for about 45 minutes, allowing sufficient time for tomograph measurements.

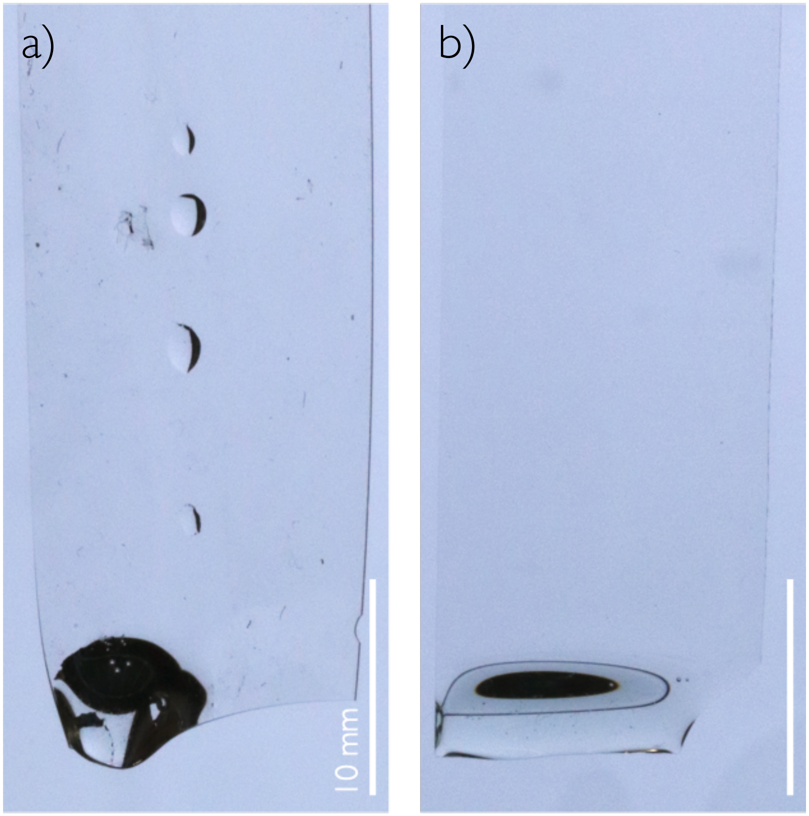

Figure S2: Pictures of ribbons a) without, b) with plasma cleaner treatment.
A plasma treatment hydrophilizes the surface of the lamella, as in b) the soap solution is homogeneously distributed over the lamella, whereas in a) drops are formed.

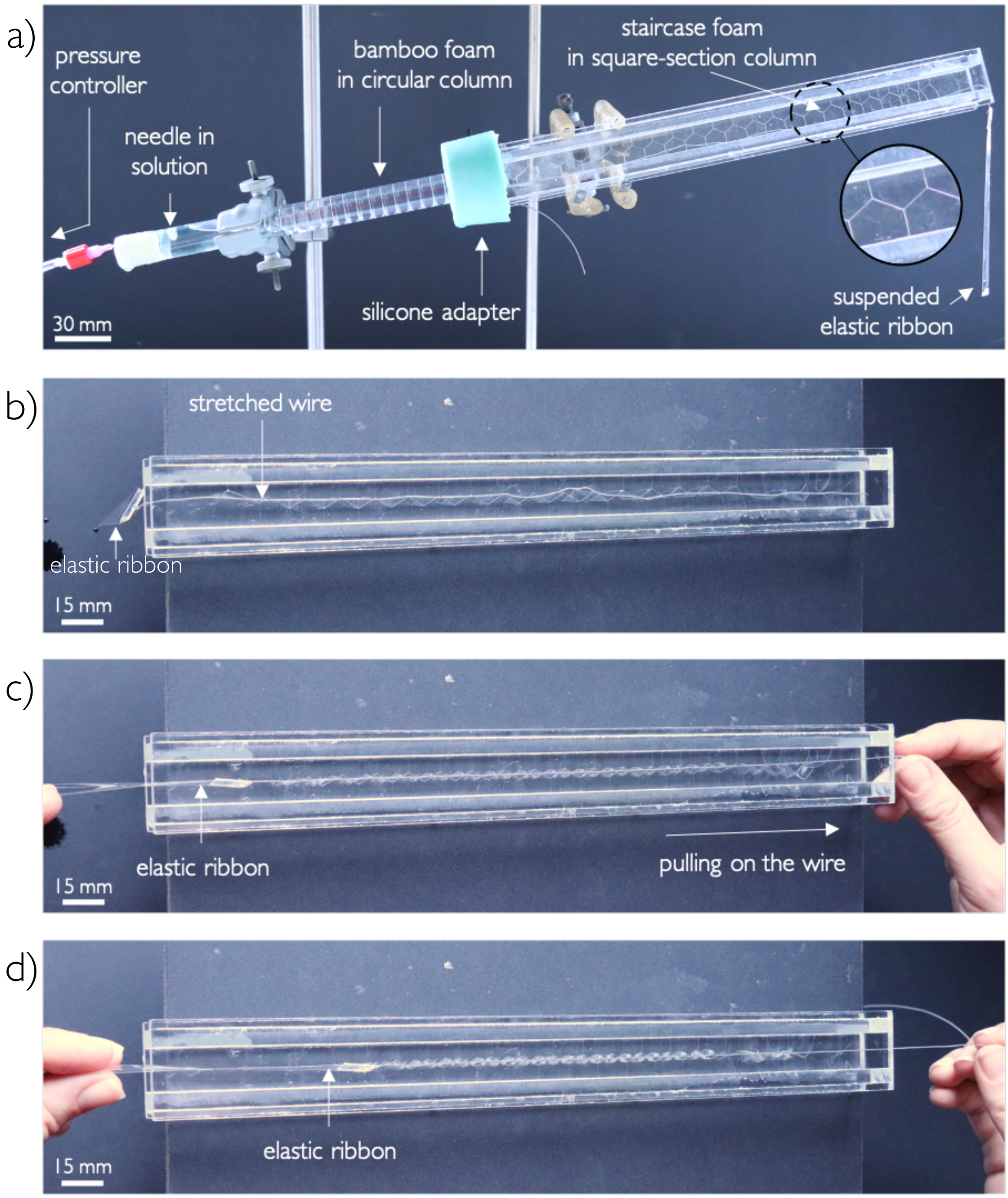

Figure S3: Pictures of our experimental setup.
a) We connect a circular tube to a PMMA square section column into which we place a stretched wire at the end of which an elastic ribbon is suspended.
b) We disconnect the square section column from the bubbling setup and place the wire in the centre of the staircase structure.
c-d) By carefully pulling on the wire, we introduce the ribbon into the central soap films.
